# Antibiotics for bronchiectasis exacerbations in children: rationale and study protocol for a randomised placebo-controlled trial

**DOI:** 10.1186/1745-6215-13-156

**Published:** 2012-08-31

**Authors:** Anne B Chang, Keith Grimwood, Colin F Robertson, Andrew C Wilson, Peter P van Asperen, Kerry-Ann F O’Grady, Theo P Sloots, Paul J Torzillo, Emily J Bailey, Gabrielle B McCallum, Ian B Masters, Catherine A Byrnes, Mark D Chatfield, Helen M Buntain, Ian M Mackay, Peter S Morris

**Affiliations:** 1 Child Health Division, Menzies School of Health Research, Charles Darwin University, Darwin, NT, Australia; 2 Queensland Children’s Respiratory Centre, Royal Children’s Hospital, Brisbane, QLD, Australia; 3 Queensland Children’s Medical Research Institute, The University of Queensland, Brisbane, QLD, Australia; 4 Queensland Paediatric Infectious Diseases Laboratory, Royal Children’s Hospital, Brisbane, QLD, Australia; 5 Department of Respiratory Medicine, Royal Children’s Hospital, Murdoch Children’s Research Institute, University of Melbourne, Melbourne, VIC, Australia; 6 Department of Respiratory Medicine, Princess Margaret Hospital, Perth, Australia; 7 Department of Respiratory Medicine, The Children’s Hospital at Westmead & Sydney Medical School, University of Sydney, Sydney, NSW, Australia; 8 Royal Prince Alfred Hospital, Sydney, Australia; 9 Department of Paediatrics, University of Auckland and Starship Children’s Hospital, Auckland, New Zealand; 10 Research and Education Support Division, Menzies School of Health Research, Charles Darwin University, Darwin, NT, Australia; 11 Department of Paediatrics, Royal Darwin Hospital, Darwin, NT, Australia

**Keywords:** Amoxicillin-clavulanic acid, Azithromycin, Bronchiectasis, Placebo, Pulmonary exacerbations, Randomised controlled trial

## Abstract

**Background:**

Despite bronchiectasis being increasingly recognised as an important cause of chronic respiratory morbidity in both indigenous and non-indigenous settings globally, high quality evidence to inform management is scarce. It is assumed that antibiotics are efficacious for all bronchiectasis exacerbations, but not all practitioners agree. Inadequately treated exacerbations may risk lung function deterioration. Our study tests the hypothesis that both oral azithromycin and amoxicillin-clavulanic acid are superior to placebo at improving resolution rates of respiratory exacerbations by day 14 in children with bronchiectasis unrelated to cystic fibrosis.

**Methods:**

We are conducting a bronchiectasis exacerbation study (BEST), which is a multicentre, randomised, double-blind, double-dummy, placebo-controlled, parallel group trial, in five centres (Brisbane, Perth, Darwin, Melbourne, Auckland). In the component of BEST presented here, 189 children fulfilling inclusion criteria are randomised (allocation-concealed) to receive amoxicillin-clavulanic acid (22.5 mg/kg twice daily) with placebo-azithromycin; azithromycin (5 mg/kg daily) with placebo-amoxicillin-clavulanic acid; or placebo-azithromycin with placebo-amoxicillin-clavulanic acid for 14 days. Clinical data and a paediatric cough-specific quality of life score are obtained at baseline, at the start and resolution of exacerbations, and at day 14. In most children, blood and deep nasal swabs are also collected at the same time points. The primary outcome is the proportion of children whose exacerbations have resolved at day 14. The main secondary outcome is the paediatric cough-specific quality of life score. Other outcomes are time to next exacerbation; requirement for hospitalisation; duration of exacerbation; and spirometry data. Descriptive viral and bacteriological data from nasal samples and blood markers will also be reported.

**Discussion:**

Effective, evidence-based management of exacerbations in people with bronchiectasis is clinically important. Yet, there are few randomised controlled trials (RCTs) in the neglected area of non-cystic fibrosis bronchiectasis. Indeed, no published RCTs addressing the treatment of bronchiectasis exacerbations in children exist. Our multicentre, double-blind RCT is designed to determine if azithromycin and amoxicillin-clavulanic acid, compared with placebo, improve symptom resolution on day 14 in children with acute respiratory exacerbations. Our planned assessment of the predictors of antibiotic response, the role of antibiotic-resistant respiratory pathogens, and whether early treatment with antibiotics affects duration and time to the next exacerbation, are also all novel.

**Trial registration:**

Australia and New Zealand Clinical Trials Register (ANZCTR) number ACTRN12612000011886.

## Background

Compared to the early 20th century, the prevalence of bronchiectasis has fallen substantially. Although regarded as an ‘orphan disease’ in affluent countries, reports of prevalence of bronchiectasis are increasing [1,2]. Bronchiectasis remains a major contributor to chronic respiratory morbidity [2,3] and mortality [1,4] in both Indigenous [5] and non-Indigenous populations [6]. In our recently completed multicentre study of children newly referred for chronic cough and managed in accordance to a standardised protocol [7], 31 (9%) of the 346 children had bronchiectasis proven on radiology [8]. In the Northern Territory in Australia, the incidence of bronchiectasis in the first year of life is 118 in 100,000 [9]. The estimated prevalence of bronchiectasis is 1,470 per 100,000 in Central Australian Indigenous children aged below 15 years [10] and 1,600 per 100,000 in Alaskan Native children [11]. In the United States, reported prevalence in adults range from 4.2 to 271.8 per 100,000 [6]. However, any reported prevalence is likely to be an underestimate as many cases are misdiagnosed or coexist with other diseases like asthma [12-14] and chronic obstructive pulmonary disease (COPD) [15]. Even without accounting for these unrecognised cases, globally there are far more patients with bronchiectasis than cystic fibrosis (CF), which has a prevalence of 7.4 to 7.9 per 100,000 in the European Union and the United States [14].

Effective clinical management reduces both short- and long-term morbidity (and likely mortality) associated with bronchiectasis [16-18]. There is increasing evidence that intensive treatment of children who either have bronchiectasis or are at risk of developing severe bronchiectasis prevents poor lung function in adulthood [17-20]. Cohort data have shown that approximately 80% of newly diagnosed adults (non-smokers) with bronchiectasis reported symptoms dating back to childhood and that the duration of chronic cough (the most common symptom of bronchiectasis [21]) was related (r = −0.51, *P* < 0.001) to lung function at diagnosis [22]. Arguably, appropriate overall management and treatment of exacerbations (leading to reduction of persistent symptoms) potentially prevents or reduces deterioration of chronic respiratory disease [23].

Determinants of accelerated lung function decline in adults with bronchiectasis are the frequency of hospitalised exacerbations, increased systemic inflammatory markers and *Pseudomonas aeruginosa* infection [24]. Amongst other factors, increased mortality risk is associated with the degree of lung function impairment [25]. No prospective data exist in children. Our study and a London-based retrospective study found that, with appropriate treatment in specialised centres, lung function improves and can be maintained [18,20]. However, those with poor lung function at diagnosis, although substantially improved, were likely to still have poor lung function five years later [20]. We also found that the only significant predictor of a decline in forced expiratory volume in one second (FEV_1_) was frequency of hospitalised exacerbations [20]. Forced expiratory volume in one second (FEV_1_)% predicted decreased by 1.95% with each previous hospitalised exacerbation [20]. As airway injury in children is superimposed upon the physiological changes involving lung growth and development [26,27], improvement in childhood bronchiectasis may impact favourably upon future adult lung function. Early and effective management of bronchiectasis exacerbations in children may lead to reduced hospitalisations, better quality of life (QOL) and improved future adult lung function.

Antibiotics are one of the key interventions used to treat acute respiratory exacerbations of bronchiectasis [21,28]. However, it is biologically plausible that antibiotics are not useful for treating some respiratory exacerbations triggered by viral infections. Our retrospective study found that 34% of exacerbations were preceded by a viral-like illness [29]. While most respiratory physicians will treat exacerbations intensively (with antibiotics and airway clearance), other doctors do not. Those choosing not to use antibiotics routinely argue that most episodes of exacerbations and cough are caused by viral infections and hence do not require antibiotic therapy. This may be appropriate but viral-bacterial interactions in the airways risk prolonged endobronchial bacterial infection that, with the associated inflammatory cascade, may cause further lung injury [23,30]. Better evidence to guide the management of exacerbations in people with bronchiectasis is needed.

### Aims of the study

There are two components of BEST; here we present the study protocol of the first phase (BEST-1). The second phase of BEST (BEST-2) will address the question “Is daily azithromycin non-inferior (within 20% margin) to amoxicillin-clavulanic acid in achieving resolution of exacerbations on day 21?” The protocol for BEST-2 will be the subject of a later paper.

This first phase of our proposed national multicentre double-blind double-dummy randomised controlled trial (RCT) is designed to answer our primary questions, as follows. Amongst children with non-CF bronchiectasis, does azithromycin improve the resolution of respiratory exacerbations by day 14 compared with placebo, and does amoxicillin-clavulanic acid improve the resolution of respiratory exacerbations by day 14 compared with placebo?

Our secondary aims are to:

1. determine the effect of azithromycin or amoxicillin-clavulanic acid on the QOL, systemic inflammation, time to next respiratory exacerbation, and duration of exacerbations;

2. explore factors that predict response to antibiotics, including respiratory pathogens (viruses, bacteria, macrolide-resistant bacteria) present in respiratory secretions and blood markers; and

3. describe the point prevalence and diversity of respiratory viruses, *Mycoplasma pneumoniae* and *Chlamydia* species during exacerbations using sensitive molecular detection techniques.

Our study tests the primary hypothesis that both oral azithromycin and amoxicillin-clavulanic acid are superior to placebo in improving the resolution rate of respiratory exacerbations by day 14 in children with non-CF bronchiectasis.

## Methods/Design

### Study design

**Figure 1:**
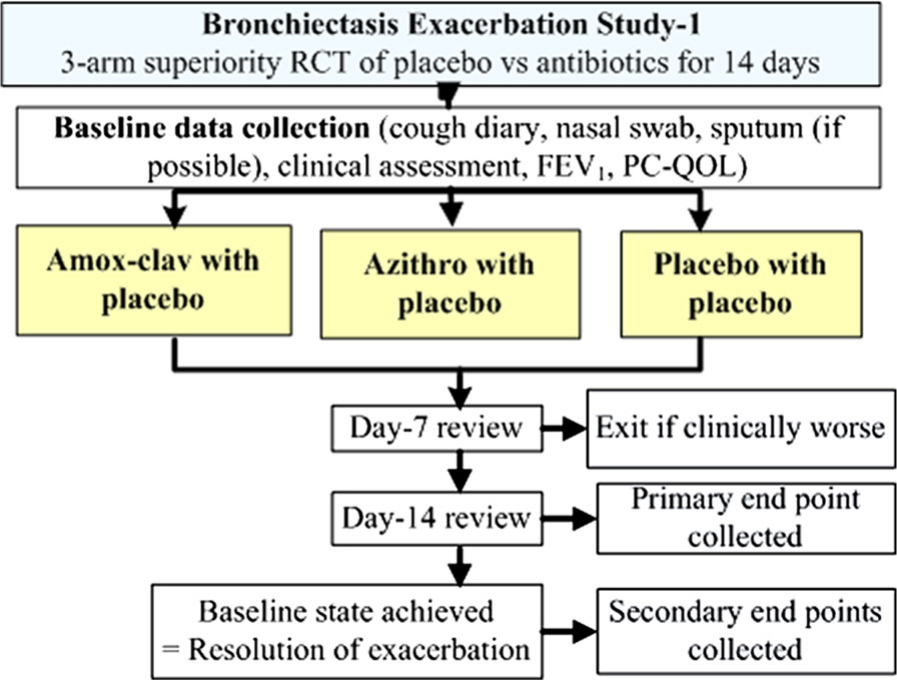
**Overall schematic study design.** Amox-clav: amoxicillin-clavulanic acid; azithro: azithromycin.

We are conducting a multicentre, parallel group, double-blind placebo RCT (with concealed allocation) to assess the impact of treatment with antibiotics (azithromycin or amoxicillin-clavulanic acid) in children with an exacerbation of bronchiectasis. Our study plan is summarised in Figure 1.

### Eligibility

The inclusion criteria are age below 18 years at time of study enrolment; diagnosed with bronchiectasis by a respiratory physician following high resolution computed tomography in the five years immediately prior to study entry or, if diagnosed earlier, have been followed regularly by a respiratory physician for treatment of bronchiectasis; and has experienced two or more respiratory exacerbations in the 18 months prior to study entry.

Exclusion criteria are current severe exacerbation of bronchiectasis (dyspnoea, hypoxia or hospitalisation), recent (in last 8 weeks) in the entry; CF; liver dysfunction; allergy or sensitivity to penicillin or macrolides; current or recent lower airway infection by a member of the Pseudomonas genus group of Gram-negative bacteria (in the four months prior to study enrolment); has received antibiotics belonging to the macrolide or penicillin class of antibiotics within three weeks immediately prior to study entry; or is currently receiving oncological treatment.

### Recruitment

Eligible children will be identified from clinics in our centres (Brisbane, Perth, Darwin and Melbourne in Australia and Auckland in New Zealand). Parents will be approached and informed consent obtained. Baseline pre-exacerbation data will be collected (Figure 1), parents contacted monthly and children reviewed every three months. Parents will be educated specifically on symptoms of exacerbations and asked to contact the research nurse at the onset of an exacerbation.

### Intervention and follow-up

A double-dummy design is planned. If eligibility is fulfilled and after informed consent has been obtained, the child is randomised to one of three arms. At the start of the exacerbation, the child will receive amoxicillin-clavulanic acid with placebo-azithromycin, azithromycin with placebo-amoxicillin-clavulanic acid or placebo-azithromycin with placebo-amoxicillin-clavulanic acid. Amoxicillin-clavulanic acid dose is 22.5 mg/kg/dose (up to 40 kg) twice a day (max 900 mg/dose). Azithromycin dose is 5 mg/kg/day, max of 200 mg daily. Equivalent volumes in placebo will be given in all arms. All treatments will continue for 14 days.

An exacerbation is defined as an increase in sputum volume or purulence, or three or more days of change in cough (> 20% increase in cough score [31] or type (dry to wet) [32]). We validated this definition in our prospective study and found that the kappa values (between clinicians) of these symptoms and signs were excellent (> 0.75) [33]. Daily diaries will also be collected during exacerbations until the scores for two or more days reflect the child’s ‘baseline’ state, which for each child will be established at enrolment, prior to any exacerbations. This assessment consists of a combination of symptoms (daily cough (yes/no), cough quality (wet/dry/none) and cough score [31] averaged over two consecutive days) and signs (sputum colour (if any present) using a colour chart card (BronkoTest Ltd, London, UK), crackles on chest auscultation). Children will be reviewed on days 7 and 14 and at resolution of the exacerbation. The exacerbation is considered ‘resolved’ when symptoms and signs are the same as ‘baseline’ state. Post exacerbation, the children will be followed-up and clinically evaluated every three months for 18 months or until their next exacerbation. ‘Time to next exacerbation’ will be determined by the number of days from ‘resolution of current exacerbation’ to beginning of the next exacerbation.

### Randomisation, allocation and blinding

Upon enrolment, the child is assigned to the next unique number on the appropriate stratified list. The allocation will be performed by the trial pharmacist at the Royal Children’s Hospital in Brisbane. Randomisation is stratified by site (Brisbane, Perth, Darwin, Melbourne, Auckland), age (≤ 5 or > 5 years) and underlying aetiology (idiopathic/post-pneumonia or all other causes). The randomisation sequence was computer generated and used permuted blocks. The allocation sequence is concealed at all times throughout the study. The computer generated allocation sequence was prepared by a statistician external to the study team.

The placebo medications, specifically manufactured by the Institute of Drug Technology Australia Limited (Melbourne, Victoria), have a similar taste and colour to their respective antibiotics. Both active medications (amoxicillin-clavulanic acid and azithromycin) are repackaged by the Institute of Drug Technology. Thus both the amoxicillin-clavulanic acid and azithromycin and their respective placebos are in identical opaque bottles. For both types of trial medications, equal volumes of water are added using a syringe and needle by punching the seal. Adherence will be assessed by parent report and return of empty bottles.

### Data collection

All data will be recorded on standardised forms. On enrolment, demographic information (age, gender, ethnicity, household size, and so on), birth history, breast feeding history, prior illness and in utero and household smoke exposure will be recorded, and a physical examination will be performed by a study physician. The primary and secondary outcome measures (see below) are collected at the time points specified above. Serious and non-serious adverse effects (nausea, vomiting, diarrhoea, rash) will also be documented and monitored. Safety exit points are discussed in End points below.

### Specimen collection

At enrolment (baseline), all children will have a deep nasal swab (NS) specimen collected. In a subset, additional specimens will be collected at baseline and during exacerbations depending on feasibility (some children are unable to attend the study centre at the onset of the exacerbation) and willingness of parents to allow additional venipuncture. These specimens are:

A deep NS specimen for respiratory viruses, respiratory bacterial pathogens (with antibiotic susceptibility testing) and other potentially important respiratory pathogens (*M. pneumoniae, Chlamydia* spp) at baseline and at the beginning and resolution of an exacerbation. The technique used is identical to previous studies [34-36] where the specimens were described as nasopharyngeal swabs. The NSs are handled as per our research laboratory protocol (see below).

Bloods at baseline and at the beginning and end of each exacerbation for C-reactive protein (CRP), neutrophilic marker of inflammation (IL-6) [37], serum amyloid A (SAA) [33,38] and markers of viral infection (interferon gamma-inducible protein 10 (IP-10) and IL-10).

Sputum at baseline and at the beginning and end of each exacerbation (when possible) for lower airway microbiology and antibiotic sensitivity.

### Further description of scores and laboratory methods

#### Cough score

The verbal categorical descriptive score is a validated daily diary score of cough rated on a six-point scale (0 = no cough to 5 = severe cough and cannot perform activities) with increasing scores reflecting greater interference with usual activities. This rating was validated against an objective cough meter measure [31] and changes in cough scores have been shown to reflect changes in objective cough counts [39].

#### Parent Chronic Cough Quality of Life score

The Parent Chronic Cough Quality of Life (PC-QOL) is a 27-item questionnaire designed to assess the level of frequency of feelings (15 items) and worry (12 items) related to their child’s cough. It uses a seven-point Likert-type scale with higher scores reflecting less frequency and fewer worry concerns (that is, greater QOL) [40,41]. The minimal important difference is 0.62 determined by the distribution method and 0.9 determined by the anchor method [42].

#### Bacteriology of nasal swabs

Oropharyngeal sampling under estimates *Streptococcus pneumoniae* carriage by approximately 50% when compared with NS [43]. Thus, NS are the preferred method when evaluating the presence of antibiotic-resistant bacteria. Culturing, identifying and, when appropriate, serotyping common respiratory bacteria are established techniques at our research laboratory [36,44]. Swabs are stored in skim milk tryptone glucose glycerol broth medium at −80°C before being batch processed for typical respiratory bacterial pathogens, notably *Haemophilus influenzae (*including strains of non-typeable *H. influenzae)*, *Moraxella catarrhalis* and *S. pneumoniae*. Batches of swabs are thawed and 10-μL aliquots cultured overnight on selective media at 37°C in 5% carbon dioxide. Growth of *S. pneumoniae*, *H. influenzae* and *M. catarrhalis* is recorded and confirmed by standard techniques [36,45]. Four isolates each of *S. pneumoniae* and *H. influenzae* and two isolates of *M. catarrhalis* per positive swab are tested for antimicrobial resistance and stored [36,45]. *S. pneumoniae* isolates are serotyped using the Quellung method (antisera from Statens Serum Institute, Copenhagen,Denmark).

In addition to routine susceptibility testing using the calibrated dichotomous susceptibility disc diffusion method, azithromycin minimum inhibitory concentration (MIC) will be determined by Etests (AB Biodisk, Solna, Sweden) if the azithromycin disc annulus is <6 mm. For *S. pneumoniae*, the penicillin MIC is determined for penicillin non-susceptible isolates (oxacillin and/or penicillin disc annulus < 6 mm) and for *H. influenzae*, the ampicillin MIC is determined for isolates if the ampicillin disc annulus is < 6 mm. Interpretive criteria (Clinical and Laboratory Standards Institute breakpoints) used for *S. pneumoniae* are penicillin non-susceptible MIC > 0.12 μg/mL, azithromycin resistance MIC ≥ 2 μg/mL; and for *H. influenzae*, ampicillin resistance MIC ≥ 4 μg/mL, azithromycin resistance MIC > 4 μg/mL. A nitrocephin-based test will identify beta-lactamase activity in *H. influenzae* and *M. catarrhalis* isolates.

#### Assessment for viruses and atypical bacteria

We will use our previous methods [46,47]. Nucleic acids will be extracted from the media using the High Pure Viral Nucleic Acid kit (Roche Diagnostics, Sydney, New South Wales, Australia), according to the manufacturer’s instructions. Real-time polymerase chain reaction assays will be used to detect respiratory syncytial viruses (A and B), adenoviruses, influenza viruses (A and B), parainfluenza, human metapneumovirus, human coronaviruses (OC43, HK1, 229E, NL63), enteroviruses, rhinoviruses (and subtypes [48]) and the more recently described human viruses (human bocavirus 1, parechoviruses, human polyomaviruses K1 and WU) and *M. pneumoniae* and *Chlamydia* species [49].

#### Blood markers

Serum CRP (threshold 5 mg/L) are standard tests that will be analysed locally (diagnostic laboratory of each participating centre). SAA, IL-6 (threshold < 3 pg/mL), IL-10 (threshold < 0.5 pg/mL) and IP-10 (threshold 2.8 pg/mL) will be performed using ELISA commercial kits (R&D Systems, Minneapolis, MN, USA) at our research laboratory.

#### Spirometry

Spirometry (in children aged ≥ 5 years) will be performed using American Thoracic Society criteria and the FEV_1_% predicted recorded. We elected not to use oscillatory measures as we found no difference in airway resistance between steady and exacerbation states [33]. Thus we will use conventional spirometry although we do not expect to detect significant differences.

### End points

Participation is complete when the child’s clinical state returns to baseline and the ‘time to next exacerbation’ has been obtained. Other exit points are if the child is clinically worse prior to day 14 or intolerance to the trial medications requiring withdrawal from the study (as determined by the treating clinician).

### Outcome measures

#### Primary outcome

The primary outcome is the proportion of children whose exacerbations have resolved by day 14. Exacerbations will be considered resolved when symptoms and signs are the same as the baseline state. Children who are withdrawn from the study or receive additional antibiotic treatment will be categorised as non-resolved.

#### Secondary clinical outcomes

The main secondary outcome is the PC-QOL score. Other outcomes are the time to next exacerbation; requirement for hospitalisation; duration of exacerbation (persistence of symptoms till ‘return to baseline state’) and FEV_1_% predicted.

#### Secondary laboratory outcomes

Serum markers (CRP, SAA, IL-6, IL-10, IP-10) and data on viruses and respiratory bacterial pathogens, including antibiotic susceptibility patterns to penicillin and azithromycin, will be the secondary laboratory outcomes.

### Sample size

We plan to enrol 189 children (63 per arm), providing 84% power (α = 0.0245, two-sided) to detect a halving of the number of children in the active arm achieving resolution by day 14 (that is, azithromycin or amoxicillin-clavulanic acid: 60% resolved by day 14, compared with placebo: 30% resolved). This is a conservative estimate when compared with our prospective data of persistent symptoms in 24% of children based on the same diary card [50]. As the primary outcome will be obtained in all enrolled children, a drop-out has not been factored in for the intention-to-treat analysis. With 20% drop-out rate, data from 153 children (51 per arm) for ‘per protocol’ analysis provides a study power of 75%. Both treatment arms are compared with the same placebo arm. While the maximum efficiency is attained by allocating more children to the placebo arm (that is, using an allocation ratio of 1:1:√2), we chose to use a 1:1:1 allocation due to ethical concerns of deviating from standard care in respiratory centres.

In the main secondary outcome (PC-QOL), based on a between-group difference of 0.9 (minimum important difference [42]) (SD 0.9), our sample size provides power of 100% (α = 0.05) for data from at least 147 children (that is, assuming at least 80% retention of the 189 children enrolled). For secondary aim 2 (exploring factors that predict response to antibiotics), we will be examining eight main factors and thus a sample size of 147 exceeds the recommended minimum (n = 10 per factor) [51]. The eight factors are smoking, age, underlying aetiology, detection of virus (any versus none, then single versus multiple viruses), presence of azithromycin resistance and blood markers (IL-6, IL-10, IP-10 levels).

### Statistical analysis and reporting

Data will be reported and presented in accordance with the updated CONSORT criteria [52]. Children will be analysed according to allocation status (regardless of subsequent management). An interim analysis is planned and a data safety and monitoring committee will determine if the study should be ceased should superiority of any antibiotic be identified after 50% of sample size is achieved.

For our primary aim, the main effects of the interventions will be determined by comparing the primary outcome (resolution of exacerbation) between groups ((azithromycin versus placebo) and (amoxicillin-clavulanic acid versus placebo)). Children who exit the study as clinically worse or drop-outs prior to the end point will be considered non-resolved. Those who exit the study as ‘returned to baseline’ will be considered resolved. Odds ratios will be calculated and, if appropriate, number needed to treat (for benefit) will be expressed. Tests of a treatment arm versus placebo at the end of the study for the primary outcome will be performed at the 2.45% significance level to account for spending some alpha at the interim analysis. Per protocol analysis will be an *a priori* secondary analysis.

#### For secondary outcomes and aims

For clinical secondary outcomes (secondary aim 1), t-tests or Mann–Whitney will be used for continuous variables (according to normality of data distribution). A Kaplan-Meier curve will be constructed for each group for ‘time to resolution’ and ‘time to next exacerbation’ as done previously [53]. For secondary aim 2 (factors that predict response to antibiotics), univariate analysis will be used to examine the biological factors listed above. Factors that have a *P-*value < 0.2 will be included in a logistic regression model. Potential interactions (for example, virus with bacteria) will be examined in the model. Descriptive data will be used for secondary aim 3 (point prevalence of respiratory pathogens).

### Data safety monitoring committee

A data safety monitoring committee has been established and has met prior to commencement of this study. It was determined that, when 50% of the sample size has been achieved, the stopping rules are as detailed below.

If superiority between each antibiotic arm and the placebo arm is shown at significance level of 0.001, the study will cease. If superiority of only one antibiotic is shown, we will continue recruiting children to the other antibiotic arm and to the placebo arm but not to the superior antibiotic arm.

If the serious adverse events (related to the medications) in each antibiotic arm outnumber the adverse events in the placebo arm at significance level of 0.01 or less, the study will cease. If increased adverse events of only one antibiotic is shown, we will continue recruiting children to the other antibiotic arm and to the placebo arm but not to the antibiotic arm related with increased adverse events. However, the study is not powered to detect between-group differences in total adverse events.

### Ethics approval

The protocol has received ethical approval from the respective Human Research Ethics Committees of all the participating institutions (Darwin: Department of Health and Families and Menzies School of Health Research; Brisbane: Children’s Health Services (Royal Children’s Hospital) and University of Queensland; Perth: Princess Margaret Hospital; Melbourne: Royal Children’s Hospital; Auckland: Northern Ethics Committee, Ministry of Health and Starship Children’s Health local ethics committee). The study is being conducted under Australia’s Therapeutic Goods Administration Clinical Trial Notification scheme.

## Discussion

Despite the considerable global burden, bronchiectasis services receive disproportionately fewer allocated resources (clinical and research) when compared with other chronic respiratory diseases [3,54,55]. The marked paucity of RCTs [21,55] is reflected in the existence of only a single (small) published placebo-controlled RCT in children with bronchiectasis [21,56]. That study described a reduction in sputum purulence and airway hyper-responsiveness in children receiving roxithromycin (n = 13) [57]. There are no RCTs on the management of bronchiectasis exacerbations in children [58]. Almost all current recommendations are based on CF management [21,28]. Such extrapolation can, on occasions, be detrimental for those with non-CF bronchiectasis. For example, a large RCT found that deoxyribonuclease (efficacious for CF) increased exacerbations and decline in FEV_1_ in adults with bronchiectasis [59] despite prior case reports advocating its use [60].

The importance of exacerbations in most chronic respiratory diseases is generally accepted. Unfortunately, data on triggers, definitions and effective treatment of bronchiectasis exacerbations in both children and adults are scarce [56,61,62]. Although viral triggers of acute exacerbations are well described in asthma [46] and COPD [63,64], no such data exist for bronchiectasis. Whether other potential respiratory pathogens (*M. pneumoniae* and *Chlamydia* species) trigger exacerbations has never been examined. Our retrospective study found that 34% of exacerbations were preceded by a viral-like illness [29]. Thus, for the first time in this population, we will determine the nature and diversity of respiratory viruses *M. pneumoniae* and *Chlamydia* species associated with bronchiectasis exacerbations.

Our study addresses a large knowledge gap in an under-researched area [55]. If the intervention is successful, it would lead to improved short-term (and possibly long-term) health benefits. Conclusive results would produce changes to evidence-based standard treatment guidelines.

### The rationale for our chosen outcome measures and timeframe

In our retrospective data of 115 respiratory exacerbations [29], we found that 35% of exacerbations failed to respond to oral antibiotic therapy (duration could not be determined) and required hospital admission. In our prospective cohort of 69 children followed for 900 child-months (156 exacerbations), 36 exacerbations (23%) were treated with intravenous antibiotics following persistence of symptoms, that is, non-resolution of the exacerbation episode. Generally, hospitalisation began three to five weeks following the initiation of oral antibiotics and ‘return to baseline’ occurred within two weeks of hospitalisation. Based on our data that 24% of otherwise well children in the community still have a cough associated with a viral infection at day 14 [50], we chose day 14 as the time point for this RCT. We also asked parents and clinicians about their willingness to use placebo for a period of time; 14 days was the limit with a safety exit point at day 7.

Adult bronchiectasis studies show that QOL measures, particularly cough-specific QOL, are valid and important outcome measures [62,65]. Likewise, we have shown the utility of a paediatric chronic cough QOL (the PC-QOL) score in children with bronchiectasis [66].

In summary, our double-blind, double-dummy RCT that examines the superiority of azithromycin and amoxicillin-clavulanic acid (compared with placebo) for exacerbations of bronchiectasis in children has the potential to have both short-term gains and a long-term benefit for reducing the morbidity of bronchiectasis.

## Trial status

We commenced recruitment in Darwin in mid-March 2012, Brisbane in June 2012 and the other sites are expected to commence in August 2012. We commenced randomisation in late May 2012.

## Abbreviations

BEST: Bronchiectasis Exacerbation Study; CF: cystic fibrosis; COPD: chronic obstructive pulmonary disease; CRP: C-reactive protein; ELISA: enzyme-linked immunosorbent assay; FEV_1_: forced expiratory volume in one second; IL: interleukin; IP-10: interferon gamma-inducible protein 10; MIC: minimum inhibitory concentration; NS: nasal swab; PC-QOL: parent chronic cough quality of life; QOL: Quality of life; RCT: randomised controlled trial; SAA: serum amyloid A; SD: standard deviation.

## Competing interests

The authors declare that they have no competing interests.

## Authors’ contributions

AC conceived the study, and participated in its design and coordination and drafted the manuscript. PM, CR, KG, PvA, AW, KO, PT and TS participated in its design and submission to the National Health and Medical Research Council. EB and GM participated in initiating the project and IMM in the viral analysis plan. IBM, CB and HB will assist in recruitment and assessment of the children. MDC advised on statistical issues. All authors read and approved the final manuscript.

## Authors’ information

ABC, CFR, ACW, PPvA, CAB, IBM and HMB are paediatric respiratory physicians, KG is a paediatric infectious disease physician, PJT is an adult respiratory physician, KFO is an epidemiologist, TPS and IMM are virologists, MDC is a statistician, EJB and GBM are research nurses and PM is a general paediatrician.
